# Application of Collagen-Based Hydrogel in Skin Wound Healing

**DOI:** 10.3390/gels9030185

**Published:** 2023-02-27

**Authors:** Yuan Zhang, Yong Wang, Ying Li, Yunpeng Yang, Mingyuan Jin, Xiaoying Lin, Zeming Zhuang, Kai Guo, Tao Zhang, Weiqiang Tan

**Affiliations:** Department of Plastic Surgery, Sir Run Run Shaw Hospital, Zhejiang University School of Medicine, 3 East Qingchun Road, Hangzhou 310016, China

**Keywords:** biopolymers, wound healing, collagen, skin repair, hydrogels

## Abstract

The repair of skin injury has always been a concern in the medical field. As a kind of biopolymer material with a special network structure and function, collagen-based hydrogel has been widely used in the field of skin injury repair. In this paper, the current research and application status of primal hydrogels in the field of skin repair in recent years are comprehensively reviewed. Starting from the structure and properties of collagen, the preparation, structural properties, and application of collagen-based hydrogels in skin injury repair are emphatically described. Meanwhile, the influences of collagen types, preparation methods, and crosslinking methods on the structural properties of hydrogels are emphatically discussed. The future and development of collagen-based hydrogels are prospected, which is expected to provide reference for the research and application of collagen-based hydrogels for skin repair in the future.

## 1. Introduction

Skin is the organ with the largest surface area and is the main barrier between the body and the external environment [1]. Loss of skin integrity due to injury or disease can lead to severe physiological imbalances such as microbial contamination, making it crucial to cover wounds after they have occurred. Wound dressings are essential in wound healing as a temporary skin substitute. Most conventional dressings used for injuries (such as gauze, bandages, etc.), despite being able to stop bleeding, absorb wound exudate, and protect against damage from bacterial infection, do not have active antibacterial and anti-inflammatory functions, and they may cause problems such as adhesions and may even hinder the wound healing process. With the in-depth research on wound healing, more demands are placed on the function of wound dressings, which serve for more than just forming a physical barrier. The ideal wound dressing will have the following characteristics: acceptable biocompatibility without producing toxicity and inflammation, good mechanical qualities to maintain the integrity of the material, adequate surface microstructure, and the biochemical capability to promote cell adhesion, proliferation, and differentiation [2]. Since 1980, dressings such as films, sponges, hydrogels, scaffolds, and nanofibers, among others, have been extensively researched. The experimental findings of Winter in 1962 revealed that moist wounds heal faster than dry ones [3].

Hydrogel materials have become ideal in wound dressing research due to their high water content, good biocompatibility, and adjustable physicochemical properties [4]. Compared with traditional dressings, such as gauze, hydrogel dressings can provide a moist environment for wound healing. By loading active substances and changing the composition and structure of hydrogels, hydrogel dressings can be endowed with excellent functions of tissue adhesion, antibacterial and antioxidant properties, and modulation of inflammatory factor expression; thus, they have a promising future in the field of wound dressing applications [4,5]. Collagen (Col), a profuse protein produced by the body, is the main structural protein found in the connective tissues, including the skin, tendons, and bones [6]. It is also the most abundant protein in the extracellular matrix [7,8]. It has a wide range of functions, including providing strength and structural stability, directing cell adhesion and migration, and regulating cellular growth and metabolism during development and repair. To date, 28 types of collagen have been identified and described; the leading fibrillar building types of collagen in the skin are I, III, and V [9]. Collagen I is the dominant form, accounting for nearly 70% of collagen in the skin. Type III is commonly found alongside type I, accounting for 15% of the skin collagen [10,11], followed by collagen IV, V, VI, and VII forming the remainder [10]. Because collagen is biocompatible, biodegradable, hemostatic, and beneficial to wound healing, adding collagen to wound dressings can enhance the critical wound healing process [7]. Hydrogels have a porous structure and a network of protofibrils, and collagen-based hydrogels facilitate cell migration and colonization, remodeling new tissue and promoting wound healing. This review summarizes hundreds of typical studies on the sources, preparation methods, and mechanisms to understand collagen-based hydrogels for wound dressings. Additionally, this review focuses on the results of in vitro and in vivo experiments based on collagen hydrogels for skin regeneration and wound healing.

## 2. Collagen

### 2.1. Roles for Collagen in Wound Healing

**Figure 1:**
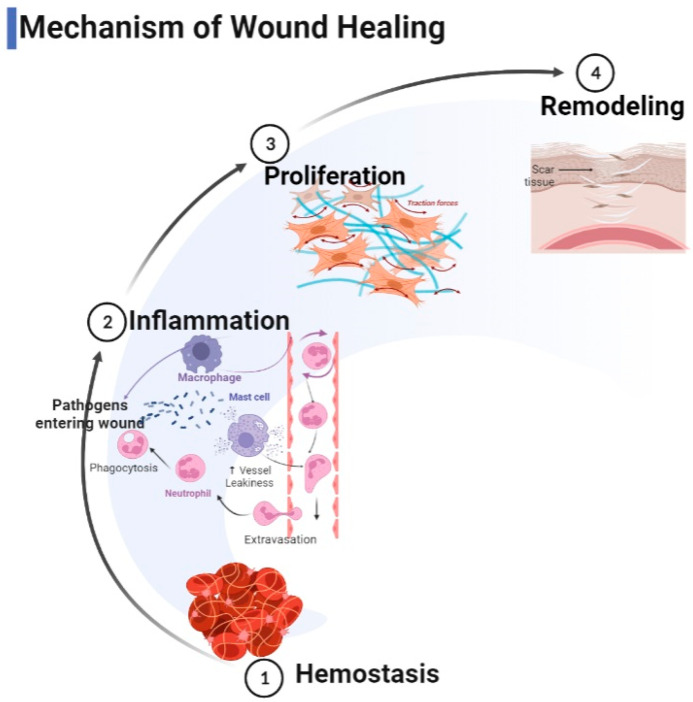
Mechanism of wound healing (produced using BioRender).

The wound healing mechanism is a complex and dynamic process that includes a series of distinct and overlapping phases, including hemostasis, inflammation, accretion, and remodeling. Collagen plays a role in each of these four stages, as schematized in Figure 1.

During the hemostatic and inflammatory phases of wound healing, collagen exposure due to injury activates the clotting cascade, resulting in a fibrin clot that stops the initial bleeding [6,8]. Collagen I and IV fragments can function as effective neutrophil chemoattractants by boosting phagocytosis and immunological responses and influencing gene expression, acting as mediators of inflammation [9,12]. Additionally, it has been shown that collagen activates microRNA signaling pathways to promote the development of a macrophage phenotype that is anti-inflammatory and pro-angiogenic. The C-propeptide fragment of collagen type I is a chemoattractant for endothelial cells [12]. This peptide fragment can play a role in the vascularization of tissues. A number of capillaries consolidate into larger vessels as the granulation matures, reducing the numbers of GAGs and proteoglycans. A collagen matrix replaces the fibronectin-rich matrix. Changes in the type, quantity, and organization of collagen also improve the tensile strength of the tissues. Type I collagen—the predominant fibrillar collagen in the skin—replaces type III collagen, which is first generated in considerable amounts. During adult wound healing, type I collagen replaces type III collagen, creating the “typical scar” [10,13].

### 2.2. Collagen Source

#### 2.2.1. Animal Source

Collagen is available from a wide range of sources, and animal collagen is currently the mainstay of collagen products, most of which are derived from raw materials such as bovine products [11]. Most collagen products are made from bovine and porcine skin and numerous fragments. One-third of the protein mass of bovine is collagen, and collagen accounts for as much as 85% of the protein in tendons. The use of animal-derived collagen is limited by religious, zoonotic, and immunological sensitivities [14]. Researchers seek a safer collagen supply to avoid BSE, TSE, FMD, and mad cow disease [15,16]. Marine sources are being researched in contrast to terrestrial animals’ unfavorable inflammatory and immunological responses and pandemic health issues [17,18,19]. Marine collagen sources are diverse, including marine vertebrates, such as fish and marine mammals, and aquatic invertebrates, such as cuttlefish, squid, octopus, shrimp, starfish, and algae, or other marine sources—especially microalgae. Currently, the research of collagen extracted from marine organisms for tissue engineering biomaterials mainly focuses on marine fish, jellyfish, sponges, etc. These aquatic organisms’ skin, muscles, and cartilage tissue are rich in collagen [20,21]. Collagen of marine origin is not only similar to traditional terrestrial mammalian collagen in terms of amino acid composition and biocompatibility, but also has greater advantages than mammalian collagen in terms of vast sources, easy extraction, absence of pathogenic microbial contamination, and stable chemical and physical properties—especially in the fish processing industry and fishing activities [22]. In fact, fish skin is commonly used for type I collagen extraction because they are not only very abundant, but also have no religious restrictions and no risk of disease transmission [19,23]. Notably, some collagens from marine sources are known to denature at temperatures lower than the average human physiological temperature. This temperature instability makes some collagen-derived biomaterials practically challenging—mainly when used for human medical applications [24].

#### 2.2.2. Recombinant Collagen

Although collagen of natural origin is widely used, it is not easy to modify. It may produce pathogenic and immune adverse reactions, and its batch-to-batch variability problems have led artificial, recombinant, synthetic collagen to become a new pathway. Recombinant collagen is obtained by using biogenetic engineering technology to splice gene fragments of collagen into suitable vectors with the help of tool enzymes and then transfer them into host cells to induce expression, which has the advantages of controlled quality, strong processability, good water-solubility, short periodicity, low rejection reaction, etc. [25]. With the development of biotechnology, the use of recombinant gene technology to obtain recombinant collagen via microbial fermentation methods has achieved great success. Many recombinant expression systems have been applied to the recombinant expression of human collagen, such as *E. coli* [26], yeast [27], animal cells, transgenic animals, and transgenic plants. The *E. coli* expression system is the most widely used protein expression system, with a low fermentation cost, short production cycle, and high efficiency. However, yeast recombinant human collagen is more similar to natural human collagen [28]. Yeast expression systems are more stable and do not produce endotoxins during purification and sterilization. In *P. pastoris*, many types of collagen have been successfully accumulated to commercially acceptable levels, making yeast-producing microorganisms the preferred method for producing recombinant collagen [29,30].

## 3. Collagen Hydrogel Formation Mechanism and Modification

**Figure 2:**
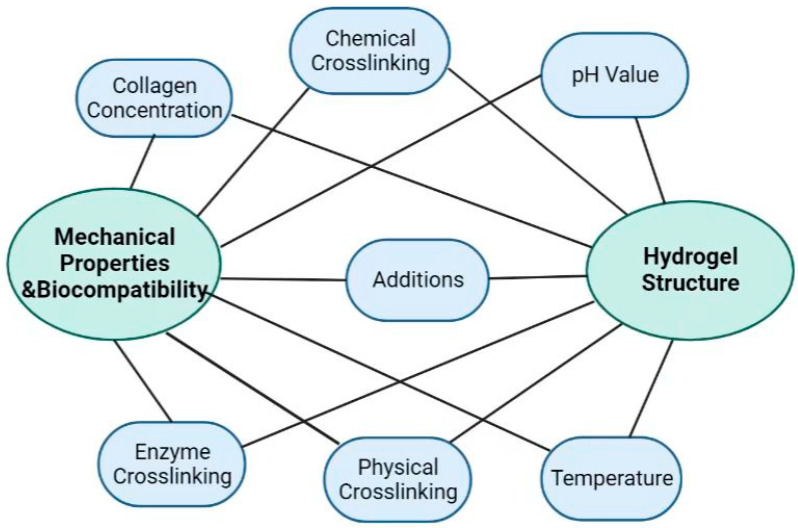
Collagen hydrogel formation mechanism (produced using BioRender).

Collagen-based hydrogels are mainly prepared with collagen as the base material. Under certain conditions, collagen molecules can form collagen fibers spontaneously and in the presence of aqueous solvents. Collagen possesses excellent mechanical stability, flexibility, and thermal and enzymatic stability throughout the body. However, when removed and utilized, qualities such as mechanical strength, thermal stability, and enzymatic degradation are drastically diminished, necessitating modification for potential biomedical applications [31,32]. The self-assembly behavior of collagen is the basis of collagen-based hydrogel processing. At the same time, changes in the solution environment, the introduction of exogenous additives, and physical or chemical crosslinking can affect the final structure and property evolution of collagen-based hydrogels by influencing the self-assembly of collagen molecules, as schematized in Figure 2.

### 3.1. Solution Condition

The solution condition (including collagen concentration, polymerization temperature, and polymerization pH) is one of the influencing factors of collagen-based hydrogels [33,34]. There is a causal relationship between the self-assembly of the collagen base and collagen concentration [35,36]. It has been shown that the mechanical properties of collagen-based hydrogels are positively correlated with collagen concentration, and as the collagen concentration increases, the pore size of collagen-based hydrogels becomes smaller, which is not conducive to cell inoculation and survival. The collagen hydrogel preparation process is closely related to the temperature [37]. Most gelation has been reported to take place at physiological temperature (37 °C), but 25 °C has also been used. In addition, the gelation process has also been performed in cryo-environments (i.e., temperatures below zero) [38]. Under higher-temperature conditions, collagen molecules denature and become faster in their self-assembly behavior, leading to a decrease in the ordered structure of collagen fibers, which ultimately affects the structure of the hydrogel. The mechanical properties of collagen-based hydrogels have been found to be positively correlated with pH. The optimal cell growth is observed in a pH range of 7.4 to 7.8. Cell growth declines precipitously as that range moves toward the alkaline side (and more gradually toward the acid side) of the optimal pH range [39,40,41].

### 3.2. Introduction of Additives

To improve the performance of collagen-based hydrogels, a second component, also known as co-blending modification, is added to compensate for the deficiencies of the collagen material by drawing on the advantages of other materials. Synthetic polymers such as polyethylene glycol and polyvinyl alcohol mixed with collagen improve the mechanical properties of the dressing but are less biocompatible [26]. Meanwhile, natural polymers such as chitosan and hyaluronic acid mixed with collagen improve the mechanical properties of the dressing and are more biocompatible. Adding natural polymeric substances to collagen scaffolds improves the mechanical properties of the stands, increases the compressive strength and swelling rate, and reduces the degradation rate [42,43,44].

### 3.3. Physical Crosslinking 

Physical crosslinking refers to the crosslinking of collagen under the physical effects of UV light, γ-ray irradiation, heating, and freeze-drying to form a three-dimensional network structure, which results in a viscoelastic gel system [45]. This can change collagen hydrogels’ mechanical characteristics and microstructure and does not require the incorporation of any chemical agent into the patient’s tissue that might cause potential harm [45]. Dehydrothermal (DHT) treatment is an easy method of crosslinking collagen molecules that involves their exposure to elevated temperatures (>90 °C) under vacuum conditions. However, DHT crosslinking leads to collagen deformation, denaturation increases with exposure time and temperature, and the DHT-induced crosslinking reaction may take several days [46]. Ultraviolet radiation can cause the formation of unpaired electrons in aromatic amino acid residues such as chromic acid and phenylalanine. The ions produced on adjacent collagen molecules by irradiation can lead to the formation of crosslinking between collagen molecules [47]. However, collagen is sensitive to ultraviolet rays. Excessively high temperature and excessively long exposure time will lead to collagen degeneration. However, crosslinking and denaturation processes confront one another during UV irradiation [48]. The final balance of these two processes affects the final mechanical properties and degradation of collagen biomaterials [48]. This complexity makes it difficult to accurately transform crosslinking, so the combination of physics and chemistry in crosslinking has attracted the attention of many researchers. This approach usually involves the reaction between the photosensitizer and UV light to produce intra- and intermolecular links within the collagen fibers. The UV–riboflavin or UV–GelMA-induced crosslinking reaction of collagen is commonly used in the treatment of skin tissue [49,50,51].

### 3.4. Chemical Crosslinking 

Among the strategies used to improve the characteristics of biomaterials, chemical crosslinking with synthetic agents is the most prevalent. Chemical crosslinking is the modification and reaction of functional groups such as carboxyl and amino groups in collagen by the selection of suitable crosslinking reagents, and the polymer chains are crosslinked to form the desired collagen hydrogel. This method allows rapid crosslinking to form a hydrogel, but the crosslinking agent can still remain on it after washing [52]. Glutaraldehyde (GA) was the first crosslinking agent utilized, due to its high reactivity and affordability. The two aldehyde groups of glutaraldehyde can form Schiff bases with two primary amino groups of the same or different molecules to link the collagen. The material’s byproducts and unreacted chemicals cannot be removed after repeated washing [52]. Reactants such as 1-ethyl-3-(3-dimethylaminopropyl) carbodiimide (EDC) and N-hydroxysuccinimide (NHS), genipin, and dialdehyde starch (DAS), which are significantly less toxic to cells, are substitutes for glutaraldehyde [53,54,55]. EDC-NHS is a “zero-length” crosslinking agent that can facilitate the formation of amide bonds by linking the carboxyl group of glutamic or aspartic acid collagen to the amino group. The crosslinking process converts EDC to water-soluble urea derivatives without releasing them into the collagen matrix. Thus, EDC/HNS has low cytotoxicity, while dramatically enhancing collagen’s physicochemical properties [56]. Genipin is a compound extracted from *Gardenia jasminoides* fruit [55]. It has a variety of active groups, such as hydroxyl and ester bonds, which can react directly with amino acids or proteins and can better maintain the basic structure of the collagen scaffold while improving its biological stability. Dialdehyde starch (DAS) is a macromolecular aldehyde formed by the reaction of natural starch and periodate. It can produce a crosslinking reaction with collagen amino and imino groups as a polyaldehyde polymer. DAS exhibits very low toxicity, is biodegradable, and has antiviral activity, and it has been used as a crosslinking agent of collagen to modify its mechanical properties [57].

### 3.5. Enzymatic Crosslinking

Compared to physical and chemical crosslinking, enzymatic crosslinking is favored for its mild reaction conditions, lack of byproducts, high specificity, and high catalytic efficiency and yield. Enzymatic crosslinking uses enzymes such as lysyl oxidase (LOX), glutamine transaminase (MTG), and horseradish peroxidase (HRP), which modify amino groups and produce protofibril bonds [58,59]. It exhibits high catalytic efficiency and favorable responsiveness to the surrounding environment. Under physiological conditions, collagen is stabilized through enzymatic post-translational modifications. This enables collagen to maintain its stability, elasticity, and biological activity. Currently, one of the commonly used enzymes that reinforce collagen’s mechanical strength is glutamine transaminase (MTG) [44]. Glutamine transaminase catalyzes the conversion of glutamine residues in collagen’s γ-hydroxylamine group and primary amine compound (acyl receptor). Then, the acyl transfer reaction generates an isopeptide bond, resulting in the covalent crosslinking of the collagen [60]. Horseradish peroxidase (HRP) is a previously commercialized and widely used plant peroxidase. HRP catalyzes phenol-rich polymers by consuming H_2_O_2_ as an oxidan [61]. Collagen contains many tyrosine residues, which the HRP-H_2_O_2_ system can oxidize to generate active free radicals and polymerize with collagen [43]. Laccase (LAC) is a multi-copper-containing oxidase. Recently, the fact that collagen can be further stabilized by crosslinking with LAC has been demonstrated [49]. 

## 4. Collagen-Based Hydrogel for Skin Wound Healing

According to recent research, the hydrogel mats for wound care can be divided into three categories: (a) pure collagen, (b) collagen blends with natural and/or synthetic polymers, and (c) collagen blends with bioactives. Various studies of collagen hydrogels are summarized in Table 1.

### 4.1. Pure Collagen

Collagen can be loaded with therapeutic agents to improve their biological outcomes; however, in some instances, they are employed as simple collagen without bioactive agent integration. Since the integration of bioactive chemicals can lead to hazardous responses [52], collagen in its purest form is safe. It has been reported that collagen-based hydrogels are excellent for use as scaffolds for wound dressings. Ge et al. extracted collagen from the skin of tilapia (PSC) and formulated a new hydrogel wound dressing containing 10 mg/mL PSC [22]. NIH-3T3 cells cultivated with the gels for 3 days displayed good biocompatibility and no toxicity in MTT cytotoxicity tests, showing that PSC hydrogels are appropriate for application on wounds. In vivo investigations revealed that the healing rate of the collagen hydrogels group was significantly higher than that of the other group on days 14, 21, and 28 [22]. Jridi et al. examined the structural and rheological properties of a collagen-based gel generated from cuttlefish skin, as well as its potential to promote wound healing [62]. Rat models treated with collagen gel for 8 days had a very quick wound closure, which may be attributable to an increase in hydroxyproline. Therefore, collagen gel has the potential to operate as a wound healing agent, since it increases extracellular matrix remodeling and speeds up the repair of tissue wounds.

### 4.2. Multi-Component Collagen-Based Hydrogels

When using hydrogels prepared with collagen alone, the wound repair capacity is much less than that of composite hydrogels with other substances added. The mechanical strength of pure collagen-based hydrogels is inferior. For application in skin tissue engineering, collagen blends with natural and synthetic polymers such as hyaluronic acid (HA) [58], chitosan, polyvinyl alcohol (PVA) [26], and polyethylene glycol (PEG) have been produced in order to improve their characteristics.

#### 4.2.1. Collagen Blends with Natural Polymers

Hyaluronic acid (HA) is the simplest glycosaminoglycan (a class of negatively charged polysaccharides) and the main component of the ECM. It participates actively in proliferation, migration, and tissue remodeling throughout the wound healing process. A shift in pore size is caused by a rise in the HA ratio, which promotes water absorption [81]. Ying et al. reported the generation of an ECM-like collagen and hyaluronic acid (Col/HA) hydrogel by using covalent crosslinking [58]. The swelling ratio of the Col/HA hydrogel was significantly greater than that of an individual Col hydrogel. It was proposed that hyaluronic acid could improve the swelling characteristics of the Col/HA hydrogel, primarily due to its high water absorption capacity. In vitro data showed that human microvascular endothelial cells (HMECs) and fibroblasts (COS-7) cultured within this hydrogel showed significant proliferation behaviors. When the scaffold was examined in vivo, the wound treated with the Col/HA hydrogel generated the thickest granulation tissue, measuring about 1300 μm, which was 300 μm thicker than the other groups. It has been demonstrated that HA crosslinking can reduce the degradation of Col/HA hydrogels, while causing only a mild inflammatory response. This hydrogel played an active role in promoting the spontaneous growth of vasculature, epithelium, and collagen fibers, as evidenced by the fast wound healing observed when it was applied to a lesion [58]. 

**Figure 3:**
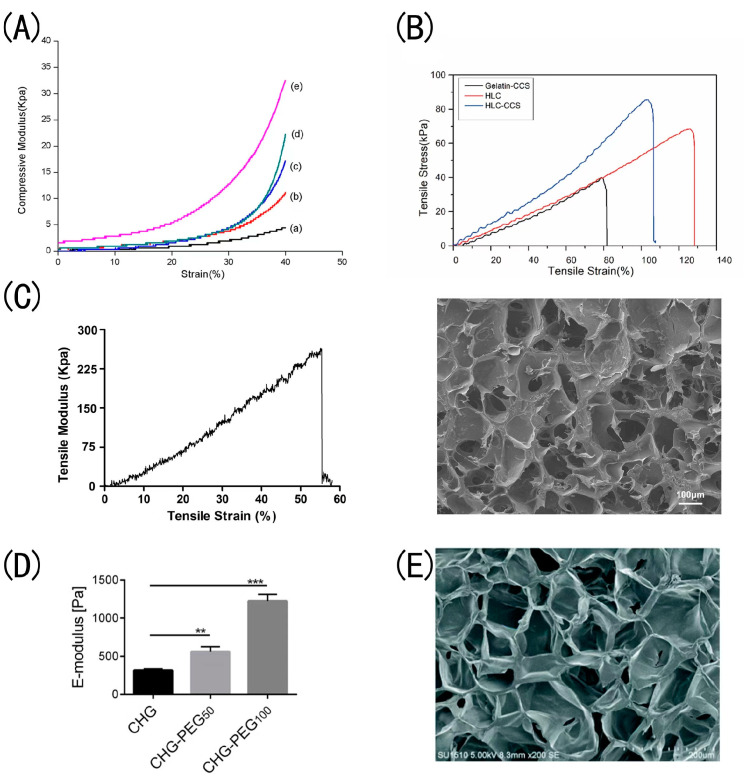
Mechanical properties of hydrogels: (**A**) Compressive modulus–strain curves of DN hydrogels with different Col:PVA ratios (reproduced with permission from Ref. [26] 2018 Elsevier B.V). (**B**) The tensile strains at the breaking points of the gelatin–CCS hydrogel, HLC hydrogel, and HLC–CCS hydrogel were 82.675%, 121.734%, and 107.2258%, respectively (Adapted with permission from Ref. [43] 2020 Elsevier B.V.). (**C**) Optimized mechanical properties and pore structure of HLC/HA/CCS hydrogel (Adapted with permission from Ref. [44] 2017 American Chemical Society). (**D**) Mechanical stability of collagen hydrogels shown as the compression E-modulus, which was calculated as the ratio of stress to strain in the linear region of the stress–strain curves in the compression tests. ** *p* < 0.01, *** *p* < 0.001 (Adapted with permission from Ref. [64] 2017 American Chemical Society). (**E**) The Col/APG hydrogel presented a uniform and dense porous structure in the SEM image. Scale bar = 100 um (Adapted with permission from Ref. [75] 2021 Royal Society of Chemistry).

Chitosan is a carbohydrate formed from chitin found in crab shells. Although it is not a component of the human ECM, the body tolerates it well. Chitosan is a desirable biomaterial for tissue engineering because it has antimicrobial characteristics [42]. Cao et al. created a hydrogel composed of human-like collagen (HLC) and carboxymethylated chitosan (CCS) using an enzyme–chemical double-crosslinking technique [43]. Compared to the control group, the HLC–CCS hydrogel exhibited the highest tensile stress (93.858 kPa) and breaking tensile modulus (112.068 kPa) (Figure 3B). HLC–CCS offered the best tensile qualities, according to the author, since HLC has a long-chain structure and can generate an exemplary network structure through crosslinking [43]. With the introduction of CCS, HLC–CCS hydrogels can also undergo crosslinking processes under the secondary crosslinking of EDC/NHS, resulting in the formation of stable chemical bonds that improve the tensile qualities related to mechanical strength. In addition, they found that HLC–CCS hydrogels promote wound healing via hemostasis, improved macrophage–fibroblast transdifferentiation, and enhanced mechanical strength [43]. Immunohistochemical results showed that HLC–CCS hydrogels can promote cells to secrete more cell growth factors (e.g., VEGF and CD31). In another work, Lei et al. synthesized a hydrogel dressing using human-like collagen, carbonylated chitosan (CCS), and hyaluronic acid [44]. The study revealed that the tensile elastic modulus of hydrogels increased with increasing HLC concentration, decreased with increasing HA or CCS concentration, and increased with increasing tensile strain and pore size (Figure 3C). The hydrophilic group of collagens was enhanced by the addition of HA and CCS. In addition, they developed a deep second-degree burn model and established that the hydrogel dressing could effectively prevent bacterial infection, as well as confirming that the hydrogel dressing promoted burn wound healing more efficiently than commercial film (*DUO DERM*). The healing rates of the dressing groups were more than 95% on day 15, whereas the healing rate of the control group was only 88.6% on day 20.

#### 4.2.2. Collagen Blends with Synthetic Polymers

Synthetic polymers do not have bioactive moieties, but they have the advantage of being highly reproducible and easy to customize for the necessary applications, as well as low immunogenicity.

Polyvinyl alcohol (PVA) has exceptional mechanical properties. PVA and collagen were used to create hydrogels by Wang et al. [26]. They discovered that the tensile mechanical properties of the hydrogels increased with the addition of PVA. Compared to pure collagen hydrogels, the stress of mixed hydrogels increased dramatically from 6 to 33 kPa at a 40% strain (Figure 3A), indicating that PVA was primarily responsible for the superior mechanical properties of the hydrogels through the creation of advanced network entanglements. Experiments including permeability, scanning electron microscopy (SEM), and water retention indicated that the microstructure of the mixed hydrogels was solid and comprised of close-knit meshes, which is consistent with the mechanical characteristics results. The potential applicability of the mixed PVA and collagen hydrogel as a wound healing scaffold was explained. In 2019, Pan et al. designed another new composite hydrogel of HLC, PVA, and sodium alginate (SA) as a wound dressing [63]. The hydrogel dressings possessed excellent hemostasis activity, with the shortest hemostasis time of 17.33 s. They observed that the PVA/HLC/SA hydrogels achieved wound closure on day 10 in a rabbit model of full-thickness wounds. Their results showed that the hydrogel could accelerate the contraction of full-thickness wounds and promote wound healing and new skin formation, showing its great potential as a dressing for skin wound healing.

The multi-armed poly(ethylene glycol) (PEG) component has amine-reactive chemistry that binds proteins/tissue and is hydrolytically degradable. In this situation, the succinimidyl ester in PEG can react with the primary amine groups in collagen, forming a crosslinked network that may have applications in filling tissue defects or facilitating wound healing. Lotz et al. chemically crosslinked type I collagen and four-armed succinimidyl glutarate poly(ethylene glycol) to form a hydrogel [64]. They found that the collagen hydrogel was more mechanically stable and less susceptible to enzymatic degradation by collagenase. The E-modulus of the crosslinked collagen hydrogel increased significantly from 315 Pa for the non-crosslinked control up to 557 Pa (Figure 3D). The collagen hydrogels could reduce fibroblast-mediated contraction. The authors observed that the crosslinked collagen hydrogels maintained their initial surface area for 21 days, while the standard skin model shrank by up to 50%, indicating that the crosslinking of collagen with PEG–SG reduced the shrinkage of collagen hydrogels. However, PEG–SG may inhibit the proliferation of dermal fibroblasts within the gels. 

Several characteristics of simple collagen-based hydrogels make them promising scaffolds for skin regeneration and wound healing applications. Collagen and other polymers (PEG, PCL, PLLA, etc.) were combined to produce a hybrid hydrogel with excellent mechanical qualities equivalent to those of human skin. All of the abovementioned features of collagen-based hydrogels expedite wound healing, especially in vivo, making them great candidates for wound dressing and skin regeneration applications. Despite the presence of these essential characteristics, these scaffolds lack the biological activities (e.g., antibacterial activity and antioxidant efficiency) required for effective skin wound dressings. The incorporation of bioactive compounds into collagen hydrogels may be utilized to treat skin wounds.

#### 4.2.3. Collagen Blends with Bioactives

Various bioactive agents can be blended into wound dressings to enhance their therapeutic effect. Antibiotics, metal nanoparticles (e.g., silver, copper), plant extracts (e.g., curcumin), growth factors, and microbes are examples of bioactive substances. Some of these therapeutic compounds can considerably enhance the hydrogel’s characteristics, and their drug delivery capability makes them ideal for wound healing.

Blending with functionalized nanoparticles is a novel technique for stabilizing and fortifying collagen tissues. Nanoparticles of metal oxides have been utilized to improve the mechanical characteristics of collagen-based scaffolds by crosslinking collagen through direct binding to its side-chain material. Using metal nanoparticles imparts the properties of metal ions to crosslinked scaffolds, such as broad-spectrum antibacterial properties, scavenging of free radicals, and reducing inflammation, in response to the rapid development of antibiotic resistance in recent years, the worsening of infections caused by drug-resistant bacteria, and the slowed rate of wound healing.

**Figure 4:**
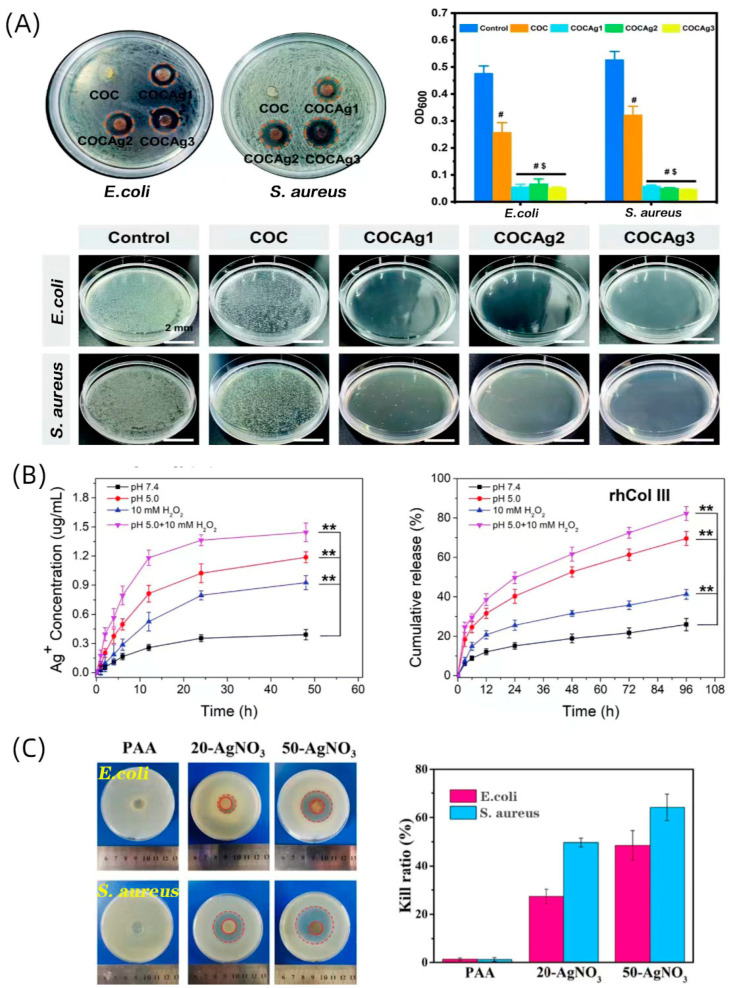
The Antibacterial properties of metal-ions-loaded hydrogel: (**A**) Inhibition zone test of hydrogels after 12 h incubation. OD values of *E. coli* solution and *S. aureus* solution after being cultured with the hydrogels for 24 h. Colony-formation images of *S. aureus* and *E. coli* on the agar plates after being treated with the hydrogels. (# *p* < 0.05 versus Control, $ *p* < 0.05 versus COC hydrogel). c (Adapted with permission from Ref. [65] 2021 Royal Society of Chemistry). (**B**) The cumulative release of Ag and rhCol III in different environments. Inhibition zone test of hydrogels after 12 h of incubation. OD values of *E. coli* solution and *S. aureus* solution after being cultured with the hydrogels for 24 h. ** *p* < 0.001 (Adapted with permission from Ref. [68] 2021 Royal Society of Chemistry). (**C**) Colony-formation images of *S. aureus* and *E. coli* on the agar plates after being treated with the hydrogels (Adapted with permission from Ref. [69] 2022 American Chemical Society).

Zhao et al. developed a collagen-based hydrogel with additional chitosan and silver ions (COCAg) [65], which exhibited exceptional antimicrobial activity. In addition, the hydrogel is injectable and has self-healing characteristics due to the Schiff base reaction. Furthermore, to improve the low stability of hydrogels, photocrosslinking combined with a Schiff base reaction was used to form a double-network crosslinked composite hydrogel. In in vivo studies, the COCAg group was observed to have the least presence of bacteria (Figure 4A). On day 14, the wounds had completely healed in the COCAg group, while those in the COC and gauze groups were still unhealed. This suggests that the COCAg hydrogel has considerable promise as a dressing for infected wounds.

**Figure 5:**
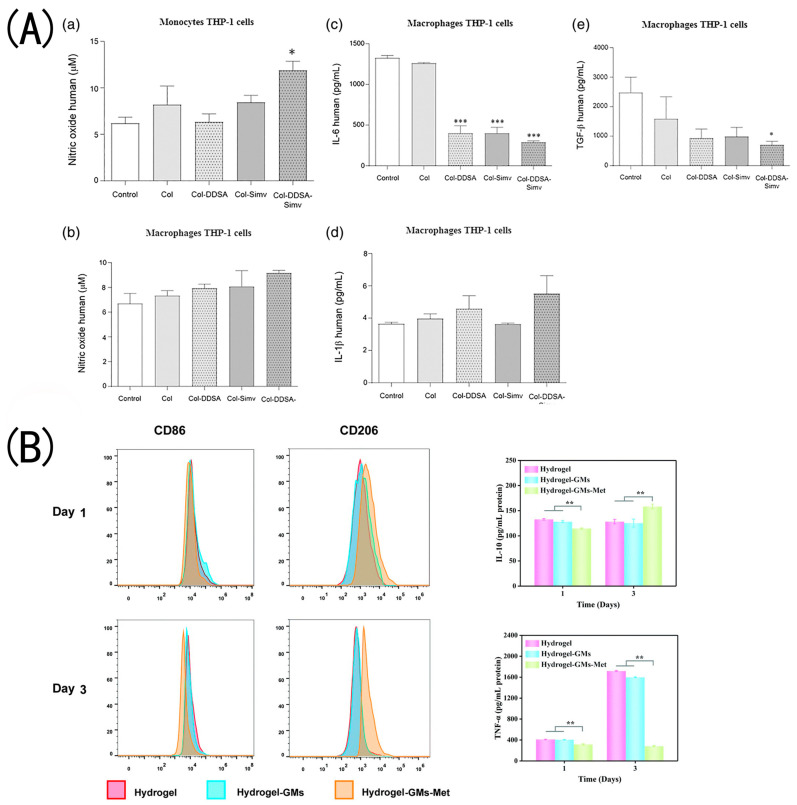

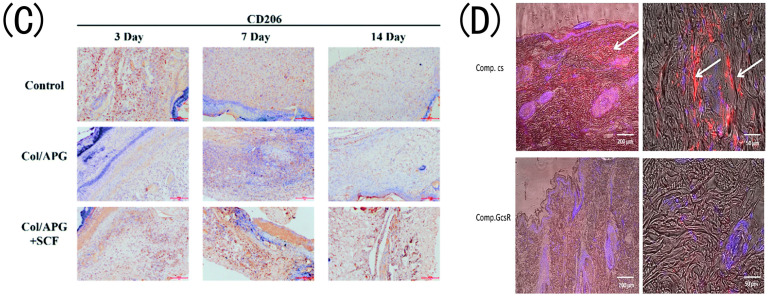
The immunoregulatory roles performed by hydrogels in inflammation alleviation and tissue regeneration: (**A**) Collagen materials loaded with simvastatin modulate the anti-inflammatory activity of macrophages. * *p* < 0.05, *** *p* < 0.001 (Adapted with permission from Ref. [74] 2019 Wiley Periodicals, Inc.). (**B**) the expression of CD206 in M2-type macrophages increased in the hydrogel–GMs–Met group, while the level of IL-10 in the hydrogel–GMs–Met group was higher than in the other two groups. ** *p* < 0.01 (Adapted with permission from Ref. [86] 2022 Royal Society of Chemistry). (**C**) CD206-positive cells always had a relatively wide and obvious distribution in the Col/APG + SCF hydrogel group compared with the other two groups, indicating the increased amount of M2 macrophages (Adapted with permission from Ref. [75] 2021 Royal Society of Chemistry). (**D**) Immunodetection of inflammatory macrophages (phenotype M1) and CD-68 (white arrows, red staining) in animals treated with unloaded (comp.cs) and double-loaded (comp.GcsR) composites. Cell nuclei are stained in blue by DAPI (Adapted with permission from Ref. [82] 2018 Elsevier B.V.).

Alvarez et al. developed collagen–silica nanocomposites capable of delivering two antibiotics [66]. They encapsulated gentamicin and rifamycin in silica nanoparticles, and then they loaded the nanoparticles into collagen hydrogels to evaluate the effectiveness of the composite hydrogels for use in a drug delivery system for the prevention of chronic wound infections. They found that the presence of a collagen network could slow down the dissolution of silica nanoparticles. Thus, gentamicin could be released consistently from the nanocomposite for more than 1 week, providing excellent antibacterial activity. In contrast, at high silica concentrations, silica particles loaded with rifamycin alter the structure of the collagen hydrogels, and the released rifamycin is adsorbed on the surface of positively charged collagen fibers, resulting in a lack of antimicrobial efficiency in the composites. Following that, the authors studied the in vivo performance of these materials intended for use in wound healing, as well as their biocompatibility and possible inflammatory reactions [82]. The results showed a 2 log steps reduction in the number of bacteria within the wound in vitro, and the associated inflammation was resolved. The histological examination of albino rabbit skin indicated the absence of M1 inflammatory macrophages in the wound bed after treatment (Figure 5D). The intricate interplay of interactions between medicines, silica, and collagen is a key factor regulating the capabilities of these composite hydrogels as antibiotic-delivering biological dressings. A collagen-based hydrogel loaded with AgNPs was formulated by Zhang et al., and the hydrogels demonstrated excellent antibacterial efficacy against *Escherichia coli* and *Staphylococcus aureus* [67]. The relative vitality of NIH3T3 cells was greater than 72% when the concentration of AgNPs in the CA–AgNPs biocomposite was less than 50 μM compared to the control group. These findings indicated that the CA–AgNPs biocomposite exhibited little cytotoxicity when the concentration of AgNPs was below 50 μM. In another study, Hu et al. prepared a hydrogel from silver-loaded polydopamine nanoparticles (PDA@AgNPs) and recombinant human collagen type III (rhCol III), with the programmable release of metallic particles. The hydrogel presented greatly improved antibacterial ability against *S. aureus* and *E. coli* after being loaded with silver. The hydrogel took advantage of boronic ester bonds to be microenvironment (i.e., pH and ROS)-responsive, leading to a controlled and programmed responsive release of therapeutic agents in which the release rate of rhCol III was significantly slower than that of silver ions in acidic and high-ROS conditions [68] (Figure 4B). As a result, the initial burst release of silver ions quickly destroyed the bacteria and reduced the inflammation, while the subsequent release of rhCol III encouraged cell proliferation and migration for the various phases. In vitro, the complex hydrogel group achieved a 98% wound healing rate in type II diabetic rats at day 14, with almost complete wound healing. Modified collagen can also be used as a polymerization catalyst [69]. Zhang et al. prepared a collagen-based hydrogel composed of pyrogallol-containing macromolecules and a transition metal (Ag), which could self-catalyze the hydrogel without external stimuli, yielding a rigid interpenetrating polymer network (IPN). At the same time, the silver ion particles conferred excellent antibacterial properties on the hydrogel (Figure 4C). Moreover, this multifunctional collagen-based hydrogel exhibited excellent hemostatic properties. More importantly, the complex hydrogel exhibited favorable conductivity and strained sensitivity as a flexible biosensor, showing that collagen-based hydrogels provide promising options for a broader range of applications.

Inflammation focuses on destroying bacteria and removing debris. Proper inflammation is required for wound healing. Excessive inflammation can result in high levels of oxidative stress and reactive oxygen species, which rapidly increase and kill cells by starting chain reactions. Therefore, hydrogels with antioxidant properties can promote wound healing. Many herbal extracts have significant antioxidant properties and can promote the wound healing process. In one study, Antezana et al. described a collagen hydrogel containing silver nanoparticles (AgNPs) and *Cannabis sativa* oil extract [70]. Due to the incorporation of AgNPs, the collagen gel displayed improved mechanical characteristics and greater resistance to collagenase breakdown. Cannabis sativa’s antioxidant characteristics can explain the increased rate of cell proliferation in materials containing *Cannabis sativa* compared to collagen hydrogels with only silver nanoparticles, indicating improved biocompatibility. In terms of antimicrobial activity, Col–CS has no inhibitory effect on the growth of *P. aeruginosa*. However, hydrogels containing AgNPs and CS did show antimicrobial activity against *S. aureus*. Bioactive agent loading (*Cannabis sativa*) increased biocompatibility, suggesting a synergistic impact of dual-drug-loaded collagen hydrogels, making these scaffolds appropriate for application as wound dressings [70]. Curcumin (Cur) is also commonly used as a bioactive agent [83]. It is a natural herbal bioactive agent with anti-inflammatory [84], antibacterial, and antioxidant properties, and Shen et al. impregnated curcumin infinite-coordination polymer nanodrugs (Cur–Fe(III) ICps) into collagen hydrogel scaffolds for continuous delivery of Cur to control inflammation [71]. This hydrogel is composed of oxidized hyaluronic acid (OHA), ε-poly-L-lysine-grafted human-like collagen (HLC-EPL), and multifunctional curcumin–Fe(III) infinite-coordination polymer nanomedicines. With the properties of wound-microenvironment-responsive drug release and burn wound adaptation, the hydrogel releases Cur in response to the acidic environment of wounds in the inflammatory phase. In vitro, the antioxidant activity of the Cur–Fe(III)–HEO hydrogel gradually increased from 91.2% to 100% with increasing Cur concentration. Curcumin-containing collagen hydrogel promoted burn wound healing through anti-inflammatory and antioxidant effects, decreased secretions, and enhanced the safety and efficacy of healing. Adding Cur could improve cell migration, and in vitro wound closure of cell mass caused by cell migration was demonstrated. The wound closures of hydrogel increased by 65.8% from 48.5%. Animal experiments showed that the closing time of burn wounds was shortened from 21 days to 9 days. Karri et al. created a novel nanohybrid scaffold [72], integrating Cur into chitosan nanoparticles (CSNPs) to enhance the stability and solubility, and impregnating the generated Cur-CSNPs into a collagen scaffold (nanohybrid scaffold) for improved tissue regeneration applications. The SEM images of the scaffolds indicated their porous architecture, with a geometry ranging from 50 to 250 um in size. It was also demonstrated that the scaffold could continually administer Cur to regulate inflammation. The drug release of 94.66 ± 5.23% from the nanohybrid scaffold at 360 h ensured effective drug release over 14 days and beyond for the treatment. They observed more fibroblasts with marked collagen synthesis in the Cur-CSNPs treatment group. The study indicates that the synergistic combination of Cur (anti-inflammatory and antioxidant) and collagen (wound healing agent as a scaffold) is a potential technique for addressing multiple pathological symptoms of diabetic wounds with improved wound healing ability [72]. A new collagen-based hydrogel using quercetin, anthocyanins, and lipoic acid as active compounds was formulated by Anghel et al. It showed significant antioxidant activity and had potential for use in transdermal drug delivery devices [73]. 

Collagen is a natural hydrophilic bioactive substance that can be combined with hydrophobic drugs as a wound dressing for drug delivery. Olivetti et al. prepared collagen hydrogels grafted with dodecenylsuccinic anhydride (DDSA) to transport the hydrophobic anti-inflammatory medication simvastatin [74]. They discovered that the modified collagen hydrogels maintained their fibrous and porous structure, exhibited higher hydrophobicity, and could serve as a delivery vehicle for a hydrophobic drug. Although cell attachment and proliferation in the scaffolds are reduced after DDSA modification, they still exhibit good cytocompatibility and the potential for cell spreading. The presence of simvastatin reduced the pro-inflammatory cytokines driving the M1 phenotype, and its antibacterial and anti-inflammatory properties made it beneficial for skin wound healing. Some studies found that metformin was able to regulate macrophages into a polarized M2 type (Figure 5A), thereby reducing inflammation in type 2 diabetic mice [85]. Jia et al. prepared HA/collagen-based hydrogels loaded with metformin microspheres [86]. In in vitro experiments, hydrogels significantly inhibited the growth activity of macrophages but did not affect fibroblast proliferation. Moreover, hydrogels containing hydrogen bonds and hydrazone bonds are pH-sensitive. Met and collagen are gradually released in an acidic environment, which reduces inflammation, changes the cell polarization from M1 type to M2 type, stimulates fibroblast migration, and promotes wound healing (Figure 5B). Polymyxin B sulfate and bacteriocin were added to a collagen-based hydrogel by Feng et al. [56]. The mixed hydrogels were shown to be effective against *E. coli* and *S. aureus*. In an in vivo test, the vascular density in wounds treated with the collagen–antibiotics (AC/OSA-PB) hydrogel dressing group was measured to be 94.8 ± 16.82/${\mathrm{mm}}^{2}$, which was significantly higher than that of the other groups. The enhanced angiogenesis in the groups with added collagen was mainly attributed to the composition of collagen. The in vivo studies revealed that the wound closure rate of AC/OSA-PB was slightly higher than that of AC/OSA on SD rats’ full-thickness wounds on day 7 and day 14.

Several cell types, such as keratinocytes, fibroblasts, endothelial cells, and macrophages, may interact with one another for the wound to heal. The production of endogenous GF, cytokines, and chemokines at the wound site impacts it. To speed up wound healing, local administration of foreign cells or cytokines has proven to be quite beneficial. Hydrogel dressings loaded with cells or cytokines enhance cell proliferation, speed up wound vascularization and re-epithelialization, and decrease the duration of wound healing. Zhang et al. developed an injectable hydrogel made of collagen and polyethylene glycol (Col/APG) and loaded with umbilical cord stem cell factor (SCF) for treating diabetic wounds [75]. As seen in the SEM photos, the Col/APG hydrogel presented a uniform and dense porous structure with a diameter of 100 nm (Figure 3E); the analysis was conducted by APG crosslinking with collagen, which enhanced the crosslinking density of the hydrogel and rendered it stable during swelling, thereby enhancing the loading and circulation of cytokines. The inclusion of APG enhanced the adhesion strength of the complexed hydrogel to 17 kPa. In MTT assays of HFF-1 fibroblasts, the overall trend was growth from day 1 to day 3, suggesting the cellular compatibility of the stem cell culture medium and the hydrogels. In CD206 immunohistochemical staining, the distribution of CD206-positive cells in the Col–APG+SCF hydrogel group was relatively extensive and prominent, indicating that the number of M2 macrophages increased (Figure 5C). In a diabetic rat model, after 14 days of hydrogel application on diabetic foot ulcer wounds, the wounds were almost completely healed and the wound inflammation was effectively improved—just 4% of wounds in the Col–APG+SCF hydrogel group remained unhealed, suggesting that Col/APG hydrogel is an ideal scaffolding material, and the Col/APG hydrogel loaded with SCF is expected to be used as a dressing for diabetic tissue regeneration [75]. In 2019, Guo et al. created a transglutaminase (TG)-crosslinked HLC hydrogel filled with basic fibroblast growth factor (bFGF) to heal skin defects [76]. According to in vivo observations, the b-FGF-containing HLC–TG hydrogel could significantly accelerate the repair and healing of skin lesions without scarring. They observed that the wound healing rate of the HLC/TG hydrogel had a valuable improvement (59%), while the HLC/TG hydrogel with the bFGF group reached 65%. The non-toxic reaction of TG crosslinked HLC hydrogels allows them to be used as a growth factor and cell carrier and implanted. The immunofluorescence of collagen I indicated that the HLC/TG hydrogels with bFGF could significantly accelerate wound epithelialization and collagen deposition, which can improve skin regeneration [76]. Hence, it has excellent applicability for clinical potential [76]. Gao et al. demonstrated that an alginate microsphere–collagen hydrogel (AMS–Col gel) improved human umbilical cord mesenchymal stem cell (HUCMSC) survival, that HUCMCs encapsulated in collagen alginate gel are viable, and that the growth factors released by HUCMCs inhibited the immune response, consequently encouraging wound healing and tissue regeneration [87]. Guo et al. seeded Fibroblasts into rHC/TG hydrogel to prepare tissue-engineered skin equivalents (TESEs) [77]. In vitro data showed that the collagen-based hydrogel had good biocompatibility, revealing that the newly formed skin tissue structure in the TESE group was more complex and consisted of abundant type I collagen, while the regenerated skin matured to a thickness of 655.3μm after surgery for 18 days. 

Extracellular vesicles (EVs), naturally secreted by cells, play an important role in intercellular communication [88]. EVs are speculated to accelerate the overall wound healing process, mainly by activating anti-inflammatory pathways such as the AKT/ERK and WNT pathways [89]. Incorporating Evs into collagen hydrogels proved to have a significant application value in skin wound healing [49]. The study found that rhCol III hydrogel can be successfully loaded with HUCMSCs-secreted EVs in order to obtain a hydrogel material with a sustained-release function. Ramírez et al. prepared collagen gels integrated with *Apis mellifera* royal jelly EVs (RJ Evs) for wound healing therapies [78]. The collagen gel was demonstrated to be an effective carrier for exosome loading, integration into collagen did not alter the EV size or integrity, and gels containing 2mg/mL collagen displayed the most stable release kinetics. From the collagen gel, the RJ EVS expelled was found to hinder *S. aureus* biofilm formation to 66.9% and displayed strong antibacterial properties.

Other functional substances, such as peptides, are also included in hydrogels to play particular roles in promoting wound healing. A cell-penetrating peptide (CPP) oligoarginine was added to the collagen–chitosan gel composite [79]. In vitro, the collagen/chitosan/CPPs gel was capable of inhibiting *S. aureus* growth and showed a good ability to heal wounds. Additionally, rats were used to assess the gel’s ability to heal wounds. In histopathological observation, more inflammatory cell infiltration was observed in the gel-treated groups, indicating that the addition of CPPs to the gel increased the inflammatory response. The wound surface healing rate for the collagen/chitosan/CPPs gel group was 98 ± 4.71% after a 14-day study on murine skin wounds. Wang et al. developed a hydrogel in that cypate-conjugated antimicrobial peptides (AMP–cypates) were added to the collagen. It promoted wound healing through its efficient antibacterial, oxygen-carrying capacity and collagen precipitation [80].

## 5. Three-Dimension Hydrogels 

Compared to conventional technologies, 3D printing is an advanced technology that can construct complex 3D structures layer by layer using computer systems. The biggest challenge for 3D printing is finding biocompatible materials that are suitable for printing and that meet the mechanical strength requirements of tissue engineering scaffolds [90]. Hydrogel materials can meet this requirement and have a promising application potential as cell-friendly materials [91]. The group of natural bio-inks is represented by protein-based bio-inks, polysaccharide bio-inks, and bio-inks based on extracellular matrix (ECM) from decellularized tissues (dECM-based) [92]. In addition, collagen is one of the most widely used biological inks, which has sparked considerable interest in tissue engineering research. Particularly in the field of skin engineering, advances have been achieved in the production of hydrogel scaffolds. However, the high rigidity of the resulting scaffold might inhibit cells from spreading, and collagen that is sticky from proliferation causes nozzle blockage. Several strategies for collagen bio-inks have been investigated, including partial crosslinking, chemical modification, or even the introduction of an additional supporting polymer network [93,94].

**Figure 6:**
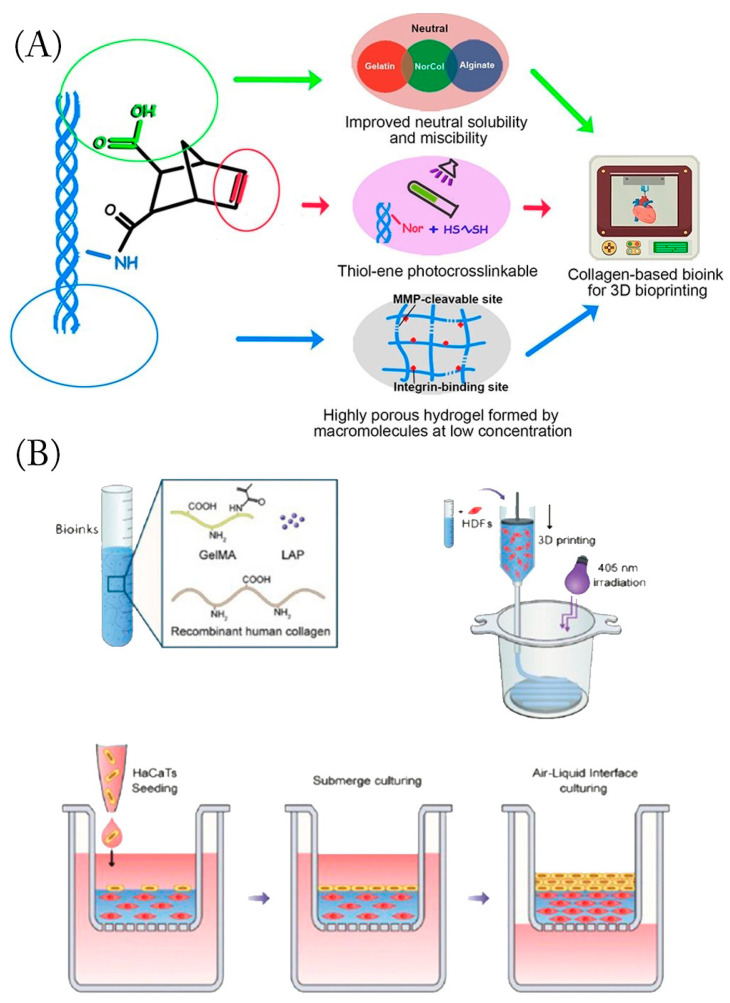

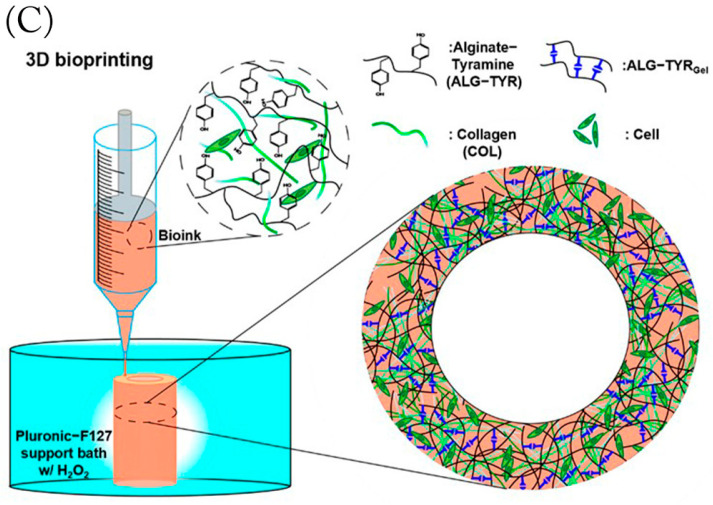
Process of making collagen bio-inks: (**A**) Molecular structure of NorCol and its principles of molecular design to improve printability and bioactivity. (Adapted with permission from Ref. [95]). 2021, American Chemical Society) (**B**) Preparation of the rhCol3-based bio-ink formulations consisting of the base component GelMA, bioactive rhCol3, and photoinitiator LAP; 3D bioprinting of the HDF-laden dermal constructs on a transwell, followed by photocrosslinking; seeding of HaCaTs on top of printed dermal constructs; submerged culturing of the in vitro 3D skin tissue; air–liquid interface culturing process to obtain the differentiated epidermal layer (Adapted with permission from Ref. [50] 2022 Yang et al.). (**C**)The bio-ink formulation contained alginate–tyramine polymer chains and collagen, which can be crosslinked to form a mechanically stable, high-resolution structure with homogeneously distributed cells (Adapted with permission from Ref [98]. 2022 Kim, S.D.).

By treating acid-soluble collagen with carbonic anhydride, Guo et al. created neutral soluble collagen (NorCol) [95] (Figure 6A). NorCol could generate hydrogels at low concentrations and did not cause clogging of print nozzles. Collagen molecules allow NorCol to form a hydrogel network with a suitable pore structure, which is the vital contributor for the excellent cell spreading and migration ability in the hydrogels. The HDFB proliferation ability was significantly higher in the NorCol hydrogels compared with the traditional collagen hydrogel. Generally, hydrogels are created by forming crosslinked networks through physical or chemical interactions. Photocrosslinking with riboflavin enables unaltered collagen hydrogels to exist in three dimensions. Collagen and riboflavin were used to create hydrogels with controlled mechanical properties mimicking those of soft tissues [96]. Diamantides et al. crosslinked collagen by activating riboflavin with blue light, substantially increasing the storage modulus of collagen bio-ink and enhancing the bio-ink’s printability [51]. 

Gelatin methacryloyl (GelMA) is usually mixed with collagen to create bio-inks for photoinitiated 3D bioprinting because of its great biocompatibility and quick photocrosslinking capabilities. Yang et al. blended recombinant human type III collagen (rhCol3) with GelMA to configure hybrid bio-inks [50] (Figure 6B). Although adding rhCol3 to GelMA slightly slows down the kinetics of thermal and photocrosslinking, as well as the mechanical properties after gelation, they successfully constructed an in vitro 3D human skin equivalent with human epidermal keratinocytes (HaCaTs) and dermal fibroblasts (HDFs) using extrusion-based 3D bioprinting. It was found that 7.5 wt% GelMA could be printed to support the stiffness of the skin without affecting the activity of the fibroblasts encapsulated within. The addition of rhCol3 to GelMA in the early stages of culture enhances the proliferation of epidermal keratin-forming cells, and the addition of rhCol3 to GelMA bio-inks facilitates faster wound healing in vivo. Effective vascularization is crucial for three-dimensional (3D)-printed hydrogel–cell constructs to efficiently supply cells with oxygen and nutrients. A lack of vascularized 3D skin substitutes may still cause skin necrosis. Stratesteffen et al. mixed GelMA with type I collagen [97]. They found that adding collagen facilitated cell migration and did not suppress angiogenesis, making it suitable for the printing of GelMA solutions containing cells. Further capillary-like network formation can be induced by co-culturing human umbilical vein endothelial cells and human mesenchymal stem cells within the proposed blends, which are appropriate for 3D bioprinting’s production of pre-vascularized tissue replacements. Additionally, 3D-printed pre-vascularized cell-laden hydrogel constructs provide a technique for angiogenesis, enabling early blood perfusion to aid wound healing.

Kim et al. synthesized an alginate/collagen hybrid hydrogel bio-ink [98] (Figure 6C) and experimentally showed that the binding of tyramine and alginate could be strengthened by adding collagen, resulting in greater polymer functionality. Their construct mimicked the ECM of a human blood vessel via the collagen bio-ink, which could be upscaled and translated for further study in vascular skin tissue regenerative applications.

In 3D printing, collagen mixed with materials is rather popular. To achieve the best printable rows, collagen and sodium alginate can be blended to obtain hydrogels with excellent mechanical and biological properties [99]. Tian et al. found that a 1% collagen composite hydrogel was shown to be a suitable bio-ink for the biofabrication of a bilayer skin construct. Fibroblasts and keratinocytes spread and proliferate in the bilayer skin [99]. In the study of Niu et al., sodium alginate/gelatin/collagen (SA/Gel/C) hydrogel as a bio-ink was used to construct a bionic full-thickness skin scaffold [100]. Huang et al. designed bionic hydrogel inks by combining gelatin and collagen. The gelatin stabilized the 3D-printed scaffold, while adding rCol III significantly accelerated wound healing and improved repair quality [101]. 

Collagen hydrogel bioprinting is not only used for skin printing for wound healing, but also used to produce cell- and organ-based systems for in vitro modeling. For example, Lee et al. deposited alternating layers of collagen, fibroblasts, and keratinocytes to produce 3D constructs that mimicked the morphological properties of native skin [102]. Moreover, a growing number of researchers are using 3D bioprinting and organ-on-a-chip (OOC) technology to reproduce the interaction between different components of the skin tissue, thereby providing a more physiologically realistic platform for testing skin reactions to cosmetic products and drugs [103].

## 6. Conclusions and Future Perspectives

Collagen is an important structural protein in the human body that promotes wound healing. The characteristics of collagen-based hydrogels include the ability to absorb and retain a large amount of water without dissolving their three-dimensional structure. The biggest advantage is that during the manufacturing process, cells and bioactive ingredients can be directly integrated into the hydrogel to obtain more favorable functions. The original single physical coverage or function of hydrogels has evolved into modern composites with numerous functionalities. Collagen hydrogels are crucial in numerous dynamic phases of wound healing, and their irreplaceable benefits make them extremely significant in wound healing and regenerative medicine. Therefore, this review summarizes the enhanced functions of hydrogel dressings co-loaded with polymers or different bioactives mentioned in the existing reports, including antibacterial, hemostatic, anti-inflammatory, and antioxidant properties, as well as substance delivery. Bacterial infection is an inevitable and vital challenge during trauma healing. However, the misuse of antibiotics has led to the emergence of multidrug-resistant bacteria, posing a more serious challenge to the antimicrobial treatment of wounds. How to achieve efficient antimicrobials and effectively promote the trauma healing process is a medical bottleneck that needs to be solved by the majority of researchers. Many antibacterial approaches have been proposed, including new medicines and their delivery systems, adding metal ions, and natural antioxidants derived from plants. Excessive inflammation usually results in the establishment of chronic wounds. Therefore, the development of hydrogels with antioxidant effects is also one of the focuses of research.

Due to wound type diversity and environmental complexity, developing and deepening further research of collagen hydrogels continuously is very necessary for better care and treatment of skin wounds. In addition to being antibacterial and antioxidant, wound dressings also have additional features (such as being injectable, self-healing, and environmentally responsive) to aid in wound healing. Three-dimensional (3D) bioprinting is also a revolutionary manufacturing process with more precise control of the fine structure of natural skin. Collagen hydrogel has the most potential as a bioprinting ink. However, due to the adverse conditions in the printing process, collagen printing leaves a lot of room for improvement, and developments in this field could dramatically improve our options for making hydrogel scaffolds and controlling their properties. Different animal models were used for the in vivo studies. Mice or rats are regularly used as model organisms, but their healing process is different from human skin, which will also lead to deviations in experimental results. Most rodent skin can heal by contracting the wound when injured. In contrast, wound healing in humans is achieved through forming granulation tissue and re-epithelialization during injury. The rabbit model is also a commonly used animal trauma model. However, this model cannot study the structural parameters observed in human proliferative scarring. Therefore, more suitable animal models also need to be explored. Animal models are appropriate for studies on wound healing; however, the intricate biochemical route involved in wound healing is not consistently represented in animal models. Thus, new approaches also need to be explored. In recent years, with the introduction of animal substitution tests in Europe and the United States and the widespread research and application of organoids, skin organs constructed in vitro have been used to simulate the human skin environment to the greatest extent possible in vitro for more intuitive and effective research and treatment of skin diseases. Moreover, 3D bioprinting technology allows the construction of more accurate in vitro skin models, and the company NOVOPLASM has successfully printed the world’s first 3D-printed immune skin model. In addition, organ-on-a-chip (OOC) is a technology developed in the past decade that can mimic human tissue and organ functions in vitro based on microfluidic chip technology. Furthermore, future research should take advantage of both, to develop 3D organ skin models that more closely resemble human physiological functions for various wound healing applications. Additionally, despite long-term research into the development of wound dressings, it is important to note that there are few approved collagen-based hydrogel wound care products. The translation of experimental materials into clinical applications is urgently required.

## Figures and Tables

**Table 1:** Collagen-based hydrogels for skin wound healing.

Source	Hydrogel Composition	Production Technique	Active Ingredient	Key Findings	Ref.
Fish	Col	Self-assembled	N	A 10 mg/mL PSC hydrogel had a proper mechanical strength In vivo data showed that the healing rate of the collagen hydrogels group was significantly higher than that of the other group on days 14, 21, and 28	Ge et al. [22]
Fish	Col	NM	N	In vivo data showed that rat models treated with collagen gel for 8 days had a very quick wound closure Histological analysis revealed that collagen enhanced wound epithelialization.	Jridi et al. [62]
NM	Col-HA	Enzymatic crosslinking	N	Material holds up the bonding and proliferation of COS-7	Ying et al. [58]
HLC	HLC-CCS	Enzyme–chemical crosslinking	N	HLC-CCS hydrogel exhibited the highest tensile stress (93.858 kPa) and breaking tensile modulus (112.068 kPa) Cytology experiments showed that HLC-CCS hydrogel is non-toxic to L929 fibroblasts and human mesenchymal stem cells HLC-CCS hydrogel can promote cells to secrete more cell growth factors (VEGF and CD31)	Cao et al. [43]
HLC	HLC-HA-CCS	Enzymatic crosslinking	N	The tensile elastic modulus of hydrogels increased with increasing HLC concentration and decreased with increasing HA or CCS concentration The healing rates of the dressing groups were more than 95% on day 15	Lei et al. [44]
Fish	Col-PVA	Double- physical crosslinking	N	The stress of DN hydrogels was greatly enhanced from 6 to 33 kPa at a strain of 40%	Wang et al. [26]
HLC	HLC-PVA-SA	Physical crosslinking	N	The hydrogel dressings possessed excellent hemostasis activity, with the shortest hemostasis time of 17.33s. A closure on day 10 in a rabbit model of full-thickness wounds.	Pan et al. [63]
NM	Col-PEG	Chemical crosslinking	N	Crosslinking of collagen hydrogels with PEG-SG reduced fibroblast-mediated contraction	Lotz et al. [64]
Calf skin	QCS-CMA	Photochemical crosslinking	N	The double-crosslinked network improved the stability of the hydrogel Collagen hydrogel loaded with sliver ions had excellent antibacterial properties	Zhao et al. [65]
Rat tails	Col	Self-assembled	NPs (gentamicin and rifamycin)	Gentamicin encapsulated in collagen–silica nanocomposite hydrogels showed sustained release from the nanocomposites over 1 week In vivo, a model showed a decrease in bacteria inside the wound by 2 log steps and resolved the associated inflammation	Alvarez et al. [66]
Bovine tendon	Col–alginate	Chemical crosslinking	AgNPs	The AgNPs-loaded hydrogel exhibited significant antimicrobial activity against both *E. coli* and *S. aureus*.	Zhang et al. [67]
rhCol	rhCol–HA	Chemical crosslinking	AgNPs	The hydrogel was microenvironment (pH and ROS)-responsive The release rate of rhCol III was significantly slower than that of silver ions in acidic and high-ROS conditions Collagen hydrogel showed a 98% wound healing rate in type II diabetic rats at day 14	Hu et al. [68]
Bovine	GCOL	Chemical crosslinking	AgNPs	Favorable conductivity and strain sensitivity as a flexible biosensor The hydrogel had excellent antibacterial activity against both *E. coli* and *S. aureus*. The hydrogel displayed superior hemostatic properties	Zhang et al. [69]
Rat tail tendons	Col	Self-assembled	AgNPs/ *Cannabis sativa* L.	*Cannabis sativa* oil improved the biocompatibility of the scaffold	Antezana et al. [70]
HLC	Col	Chemical crosslinking	Cur/Fe(III) NPs	The drug release is environment-sensitive to the pH value The antioxidant activity of the Cur-Fe (III)-HEO hydrogel increased from 91.2% to 100% with increasing Cur concentration The closure period of full-thickness burn wounds was shortened to 9 days	Shen et al. [71]
Porcine	Col–CS	Freeze-drying	Curcumin	Drug release of 94.66 ± 5.23% from the nanohybrid scaffold at 360 h In vivo data showed more fibroblasts with marked collagen synthesis after 15 days of treatment with curcumin	Karri et al. [72]
Bovine	Col–SA–cellulose	Freeze-drying	Quercetin/anthocyanins/lipoic acid	The resistance to the deformation and the elastic modulus of the cellulose–collagen–Na alginate matrix was enhanced as one or more bioactive compounds were added	Anghel et al. [73]
Rat tails	DSSA	Self-assembled	Simvastatin	DSSA hydrogel could serve as a delivery vehicle for a hydrophobic drug	Olivetti et al. [74]
Fish	Col–OSA	Chemical crosslinking	Polymyxin B sulfate/bacitracin	Hybrid hydrogel can effectively protect against *E. coli* and *S. aureus* The wound closure rate of AC/OSA-PB was slightly higher than that of AC/OSA.	Feng et al. [56]
NM	Col–APG	Chemical crosslinking	SCF	The injectable Col/APG hydrogel had good cytocompatibility In vivo studies showed that the wound was almost completely healed when treated with Col/APG +SCF	Zhang et al. [75]
HLC	Col–TG	Enzymatic crosslinking	bFGF	The wound healing rate of the HLC/TG hydrogel with the bFGF group reached 65%	Guo et al. [76]
rHC	rHC/TG	Chemical crosslinking	Fibroblasts	In vitro data showed that the collagen-based hydrogel had good biocompatibility More abundant collagen- content was observed in vivo	Guo et al. [77]
NM	Col	Self-assembled	royal jelly EVs	RJ EVs expelled were found to hinder *S. aureus* biofilm formation to 66.9%	Ramírez et al. [78]
NM	Col–CA	Freeze-drying	CCP	The addition of CPPs to the gel increased the inflammatory response	Li et al. [79]
rhCol	Col–gelatin	Enzymatic crosslinking	AC	AMP-cypates exhibited outstanding antibacterial activity, jointly achieved through antimicrobial peptide (AMP) activity, photothermal therapy (PTT), and photodynamic therapy (PDT)	Wang et al. [80]

Abbreviations: collagen (Col), none (N), not mentioned (NM), human-like collagen (HLC), carboxylated chitosan (CCS), polyvinyl alcohol (PVA), sodium alginate (SA), poly(ethylene glycol) (PEG), quaternized chitosan (QCS), methacrylate-anhydride-modified collagen (CMA), nanoparticles (NPs), silver nanoparticles (AgNPs), recombinant humanized collagen (rhCol), chitosan (CS), GA-modified collagen (GCOL), curcumin (Cur), dodecenylsuccinic-anhydride-modified collagen (DSSA), oxidized sodium alginate (OSA), polyethylene glycol (APG), stem cell factor (SCF), transglutaminase (TG), basic fibroblast growth factor (bFGF), cell-penetrating peptide (CCP), cypate-coupled antimicrobial peptide (AC).

## Data Availability

Not applicable.
